# Identification of the largest non-essential regions of the C-terminal portion in 3A protein of foot-and-mouth disease virus for replication in cell culture

**DOI:** 10.1186/s12985-020-01379-x

**Published:** 2020-09-14

**Authors:** Pinghua Li, Xueqing Ma, Xingwen Bai, Pu Sun, Hong Yuan, Yimei Cao, Kun Li, Huifang Bao, Yuanfang Fu, Jing Zhang, Yingli Chen, Dong Li, Zhiyong Li, Zengjun Lu, Zaixin Liu

**Affiliations:** State Key Laboratory of Veterinary Etiological Biology, National Foot and Mouth Disease Reference Laboratory, Key Laboratory of Animal Virology of Ministry of Agriculture, Lanzhou Veterinary Research Institute, Chinese Academy of Agricultural Sciences, No. 1 Xujiaping, Yanchangbao, Lanzhou, Gansu 730046 PR China

**Keywords:** Foot-and-mouth disease virus, 3A protein, Non-essential region, Deletion mutant

## Abstract

**Background:**

Recent study has shown that the C-terminal portion of 3A (amino acids (aa) 81–153) is not essential for foot-and-mouth disease virus replication in cell culture, however, the complete C-terminal portion (aa 77–153) of 3A is highly variable and prone to occur deletions and mutations, therefore, we presume that this region plays a very limited role and probablely is completely nonessential for virus viability.

**Methods:**

In this study, to identify the largest non-essential region of the C-terminal portion in 3A for FMDV viability, several deletions containing aa 80–153, 77–153 and 76–153 of 3A protein were introduced into an FMDV full-length infectious cDNA clone pOFS by the overlapping extension PCR. Additionally, to explore the importance of the highly conserved residue 76 L of 3A for the FMDV of Cathay topotype, two mutants containing 3A L76I and 3A L76V were generated based on the 3A deletion mutant by point mutation. We also introduced the enhanced green fluorescent protein (eGFP) into one of the 3A deletion mutants by the extension PCR to investigate the genetic flexibility of 3A to express foreign genes. All linearized full plasmids were transfected into BSR/T7 cells to rescue infectious foot-and-mouth disease viruses. The rescused viruses were analyzed by RT-PCR, nucleotide sequencing, immunofluorescence assay and western blot and were characterized by plaque assays and one-step growth kinetics.

**Results:**

The results demonstrated that the deletion of aa 80–153 and aa 77–153 and the substitutions of 3A L76I and 3A L76V did not affect the production of infectious virus, while the fusion of the eGFP gene to the C-terminus of 3A resulted in nonviable FMDV.

**Conclusions:**

Our results firstly reported that the aa 77–153 rather than aa 81–153 of 3A protein was dispensable for FMDV replication in cell culture. This study is of great significance for development of FMD marker vaccine and foreign gene expression in the future.

## Introduction

Foot-and-mouth disease (FMD) is a highly contagious disease of cloven-hoofed animals, including domesticated ruminants and pigs, as well as a large number of wildlife animals. The disease is endemic in many countries of the world and has devastating economic impact on livestock health, production and international trade. Therefore, the disease remains a major economic concern and a continuous threat for development animal husbandry worldwide [1]. The causal agent, FMD virus (FMDV), is a member of the genus *Aphthovirus* in the family *Picornaviridae* and exists as seven distinct serotypes, including A, O, C, Asia-1, and South African Territories (SATs) 1 to 3, as well as multitudes of antigenic variants [2, 3]. The viral genome is a single-stranded, positive-sense RNA of approximately 8,5 kb in length and encodes a single polyprotein that is subsequently processed by virus-encoded proteases to yield four individual structural proteins (VP4, VP2, VP3, and VP1) and ten nonstructural proteins (Lpro, 2A, 2B, 2C, 3A, 3B1–3, 3Cpro, and 3Dpol) [4, 5]. The structural proteins are needed for formation of mature virion, and the non-structural proteins are involved in the replication of viral RNA [6, 7].

Among the nonstructural proteins, 3A is 153 aa in most FMDVs examined to date and is considerably long compared to the corresponding region of other picornaviruses [8]. 3A protein contains a highly conserved N-terminal domain (aa 1–76) which contains an approximately 18 aa hydrophobic domain (HR, spanning residues 59 to 76) involved in binding membrane and a strikingly variable C-terminal portion (aa 77–153). There is no deletions were identified in the N-terminal region, however, there are substantial deletions and substitutions found in the C-terminal portion. Some deletions in 3A protein are associated with host range and virulence of the FMDV [9–11].

It has been shown that natural and egg-adapted isolates have different deletions (such as aa 92–103, 133–143, 84–102 and 88–106) at the C-terminal coding region of 3A [8–12], indicating that these deletions are non-essential for FMDV infectivity. Previous works also reported that wild FMDV strains can tolerate artificial deletions of aa 93–102, 93–143, 91–104, 87–106 in 3A [13–17]. Recent observation further revealed that the C-terminal region (aa 81–153) of 3A containing the critical residues involved in the formation of the 3A-3B1 cleavage junction, is dispensable for foot-and-mouth disease virus replication in cell culture [18]. While this study firstly showed that FMDV can tolerate 73 aa deletion in 3A, the significance of the complete C-terminal region (aa 77–153) of 3A for FMDV replication and infectious virus production is poorly understood.

In this study, to identify the largest non-essential region in FMDV 3A for viral replication in cell culture, several deletions (aa 80–153, 77–153 and 76–153) in 3A protein were introduced into an FMDV full-length infectious cDNA clone, pOFS. Additionally, multiple sequence alignment of FMDV 3A proteins indicated that the residue 76 L of 3A is highly conserved among the isolates of Cathay topotype. To investigate the importance of this conserved residue for the FMDV of Cathay topotype, two mutants containing 3A L76I and 3A L76V were generated based on the construct containing the deletion of aa 77–153 in 3A. The infectious FMDVs were recovered and the characterization and the effects of the deletions and the substitutions in 3A protein on FMDV replication were then evaluated. Finally, we also assessed the flexible nature of 3A by expressing the enhance green fluorescent protein (eGFP) in one of the 3A deletion mutants.

## Materials and methods

### Cells, plasmids and virus

Baby hamster kidney (BHK) cells were grown in Dulbecco’s modified Eagle’s medium (DMEM) (Invitrogen) containing 10% fetal calf serum (FCS) and antibiotics (100 units/ml of penicillin, 20 μg/ml of streptomycin). BSR/T7 cells were provided by K. K. Conzelmann [19] and maintained in Glasgow minimal essential medium (GMEM, Invitrogen) supplemented with 4% tryptose phosphate broth, 10% fetal calf serum (FCS) and G418 (1 mg/mL). A genome-length infectious cDNA clone (pOFS) of FMDV O/HN/CHA/93 strain has been described previously [20]. r-HN, a genetically engineered FMDV derived from full-length infectious clone pOFS, was used as a wild-type (wt) control in all experiments [20].

### Introduction of deletions into FMDV 3A protein

**Fig. 1:**
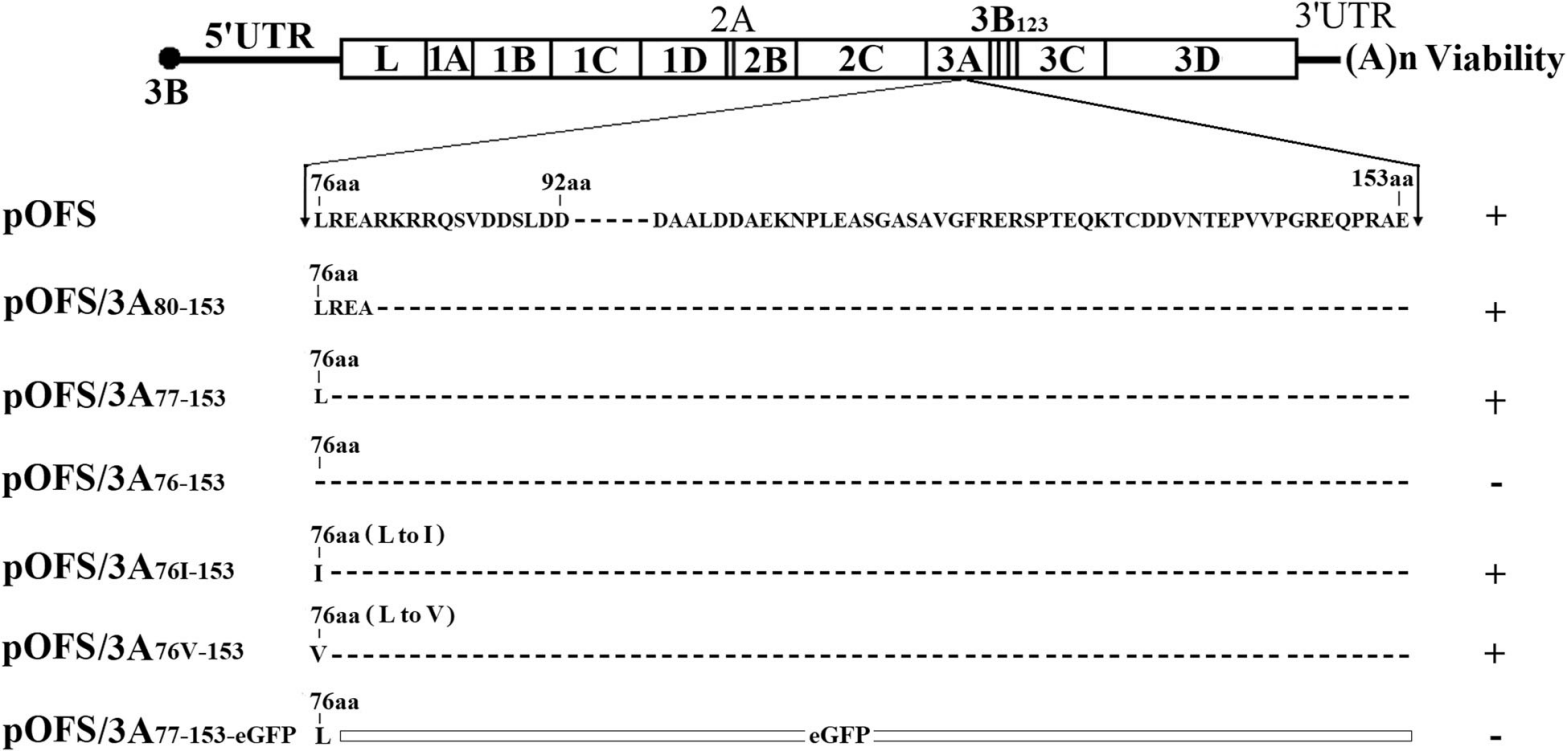
Schematic diagram of the FMDV genome and plasmids used in the present study. The mutant plasmids derived from the FMDV full-length infectious cDNA clone, pOFS. The deletions and amino acid mutations in the 3A protein were introduced by overlapping PCR. The deleted amino acids are shown as a dashed line, and the relative positions are indicated in the individual mutant. The viability for each deleted mutant is shown on the right: +, viable; −, nonviable

The C-terminal deletion mutants of FMDV 3A protein were constructed based on the full length clone pOFS. Firstly, six PCR primers (Table 1) were used to amplify three overlapping cDNA fragments containing the corresponding deletion regions as previously described [21]. Then, the resulting PCR products were digested with *Bgl* II and *Nru* I and then cloned into pOFS with the same enzymes to result in the mutant constructs of pOFS/3A_80–153_, pOFS/3A_77–153_ and pOFS/3A_76–153_. In addition, four PCR primers (Table 1) were used to amplify two overlapping cDNA fragments and two additional clones pOFS/3A_76I-153_ and pOFS/3A_76V-153_ containing the deletion of aa 77–153 in 3A and the substitutions of 3A L76I and 3A L76V were generated as above described. All full-length mutant clones were confirmed by nucleotide sequencing. Figure 1 illustrates the strategy for the construction of the deletion mutants.

**Table 1:** Oligonucleotide primers used in this study

Primer	Sequence(5′ → 3′)	usage
HN-1F	CAAGAAGTGATTGAGCGGGT	
3A_76–153_F	TGGCAAACATAGTGATCATGGGACCCTACGCCGGGCCACT	△76–153
3A_76–153_R	AGTGGCCCGGCGTAGGGTCCCATGATCACTATGTTTGCCA	△76–153
3A_77–153_F	CAAACATAGTGATCATGCTAGGACCCTACGCCGGGCCACT	△77–153
3A_77–153_R	AGTGGCCCGGCGTAGGGTCCTAGCATGATCACTATGTTTG	△77–153
3A_80–153_F	TGATCATGCTACGCGAAGCGGGACCCTACGCCGGGCCACT	△80–153
3A_80–153_R	AGTGGCCCGGCGTAGGGTCCCGCTTCGCGTAGCATGATCA	△80–153
3A_76V-153_F	CAAACATAGTGATCATGGTAGGACCCTACGCCGGGCCACT	△76 V-153
3A_76V-153_R	AGTGGCCCGGCGTAGGGTCCTACCATGATCACTATGTTTG	△76 V-153
3A_76I-153_F	CAAACATAGTGATCATGATAGGACCCTACGCCGGGCCACT	△76I-153
3A_76I-153_R	AGTGGCCCGGCGTAGGGTCCTATCATGATCACTATGTTTG	△76I-153
3A-GFP1F	AAGCGCGCAAGAGGCGCCAGATGGTGAGCAAGGGCGAGGA	▽GFP
3A-GFP1R	TCCTCGCCCTTGCTCACCATCTGGCGCCTCTTGCGCGCTT	▽GFP
3A-GFP2F	GCATGGACGAGCTGTACAAGGGACCCTACGCCGGGCCACT	▽GFP
3A-GFP2R	AGTGGCCCGGCGTAGGGTCCCTTGTACAGCTCGTCCATGC	▽GFP
HN-4R	GTTCCCTTCTTCATTCTCGC	

△indicates amino acids deletion. ▽indicates amino acids insertion

### Introduction of the eGFP gene into the 3A deletion mutant

The identification of the largest nonessential regions indicates that 3A is genetically flexible, to examine if 3A could tolerate foreign gene in the largest nonessential region, the eGFP gene omitting the stop codon (714 bp) was inserted into one of the mutant construct (Fig.1). The insertion of eGFP into the 3A deletion site was performed via three separate PCRs using six primers (Table 1) as previously described [21]. Then, the eGFP fusion fragment was generated by the overlapping extension PCR with all three fragments and the primer pair HN-1F/HN-4R. Then this fragment was excised with *Bgl* II and *Nru* I and subcloned into pOFS to result in the plasmid pOFS/3A_77–153-GFP_. Introduction of the eGFP gene into the 3A deletion mutant was confirmed by DNA sequencing.

### Transfection and recovery of the mutant viruses

All full-length plasmids were linearized with *Not* I and then were purified using PCR purification kit (Qiagen). Purified DNA was quantified by spectrophotometry at the optical density at 260 nm (OD260). For transfection, 2 × 10^6^ BSR-T7/5 cells (seeded in six-well plate) were transfected with 2 μg purified DNAs and 20 μl Lipofectamine™ 2000 (Invitrogen) according to the manufacturer’s protocol. After 5 h of incubation at 37 °C, the complete medium was added and the cells were further incubated at 37 °C, 5% CO_2_. The transfected cells were observed daily for appearance of cytopathic effect (CPE) and eGFP autofluorescence. After 72 h post-transfection, the culture supernatants were collected and passaged on BHK-21 cells. At least four further passages were performed if no visible CPE was observed after transfection.

### RT-PCR and nucleotide sequencing

The cell culture supernatants from transfected and viral infected cells (P5) were harvested. RNAs were extracted with viral RNA mini kit (Qiagen). First-strand cDNAs were synthesized using reverse transcriptase XL (AMV) (Takara) and anti-sense primer HN-4R (Table 1). The fragments containing 3A gene of the mutant and wt viruses were amplified by PrimeSTAR HS DNA Polymerase (Takara) using primer pairs HN-1F and HN-4R. The resulting PCR fragments were purified and sequenced to confirm the presence of the expected modifications.

### Immunofluorescent assay

BHK-21 cells grown on 12-well plates were infected with the transfected supernatants and wt virus. Immunofluorescence was performed at 6 h post-infection as previously described [15]. The cells were immunostained with primary antibodies (FMDV 3A and 3B monoclonal antibody) and secondary antibody (FITC-conjugated goat anti-mouse IgG) as described previously [22]. The nuclear DNAs were stained with DAPI (4′, 6-diamidino-2-phenylindole). The images were visualized under a laser scanning confocal microscope (Leica TCS SP8, Germany).

### Western blot analysis

Cell lysates from infected BHK-21 cells were prepared and fractioned on a 12% SDS-PAGE. Proteins were transferred to PVDF membranes. Membranes were blocked with dried skimmed milk in PBS containing 0.5% Tween 20 for overnight at 4 °C and incubated with primary antibodies (FMDV 3B monoclonal antibody and anti-GFP antibody) and secondary antibody (HRP-conjugated goat anti-mouse IgG) respectively. The protein bands were detected using the ECL kit according to the manufacturer’s directions.

### Plaque assays and one-step growth kinetics

Mutant viruses were characterized by plaque assays in BHK-21, FPK and PBK cells as following: Confluent cells seeded in six-well plates were infected with 200 ul serial dilutions of viral samples. After 1 h incubation at 37 °C, the cell monolayer was washed with PBS (PH 7.4) and overlaid with 2 × MEM (Invitrogen) supplemented with 2% FBS and 0.6% gum tragacanth. After 48 h incubation, the cells were then fixed in 50% acetone and 50% methyl alcohol and plaques were visualized by staining with 1% crystal violet. One-step growth analyses of the viruses were performed as described previously [14]. At different times post-infection (4, 8, 12, 16, and 20 h), cells were harvested and viruses were released from the cells by two freeze/thaw cycle. The virus titers were determined by TCID_50_ on BHK-21 and FPK cells.

## Results

### The aa 77–153 of 3A protein was dispensable for FMDV replication in cell culture and the residue 76 is essential for FMDV viability

**Fig. 2:**
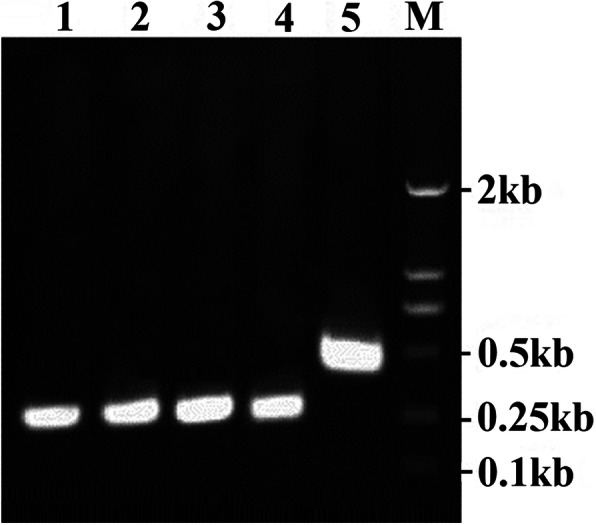
Identification of the 3A mutant viruses by RT-PCR. HN-1F/HN-4R was used to confirm the presence of the engineered deletions with the expected size in the mutant viruses. The detection of r/HN/3A_77–153_, r/HN/3A_76I-153_, r/HN/3A_76V-153_, r/HN/3A_80–153_ and r/HN viruses are shown in lanes 1, 2, 3, 4 and 5, respectively, M refers to a 2000 bp DNA ladder (Takara Biolabs)

To determine whether the remarkably variable region (aa 77–153) of 3A plays little role for the production of infectious FMDV, three full-length mutant clones (pOFS/3A_80–153_, pOFS/3A_77–153_ and pOFS/3A_76–153_) were created by over-lapping PCR fragments flanked by unique restriction enzymes. Then, these plasmids were transfected into BSR/T7 cells. After 56 h post-transfection, the cells transfected with pOFS/3A_80–153_ and pOFS/3A_77–153_ showed obvious CPE and two mutant viruses named r/HN/3A_77–153_ and r/HN/3A_80–153_ were successfully rescued. In contrast, infectious FMDV was not able to recover even after several transfections with pOFS/3A_76–153_ construct and blind passage of transfected supernatants in BHK-21 cell, indicating that the deletion of aa 76–153 of 3A is lethal to the virus. RT-PCR (Fig. 2) and sequence analysis of the rescued viruses indicated the presence of the expected deletion sequence. Taken together, these results revealed that the aa 77–153 rather than the aa 81–153 of 3A was dispensable for FMDV replication in cell culture and the residue 76 is essential for completion of FMDV life cycle.

**Fig. 3:**
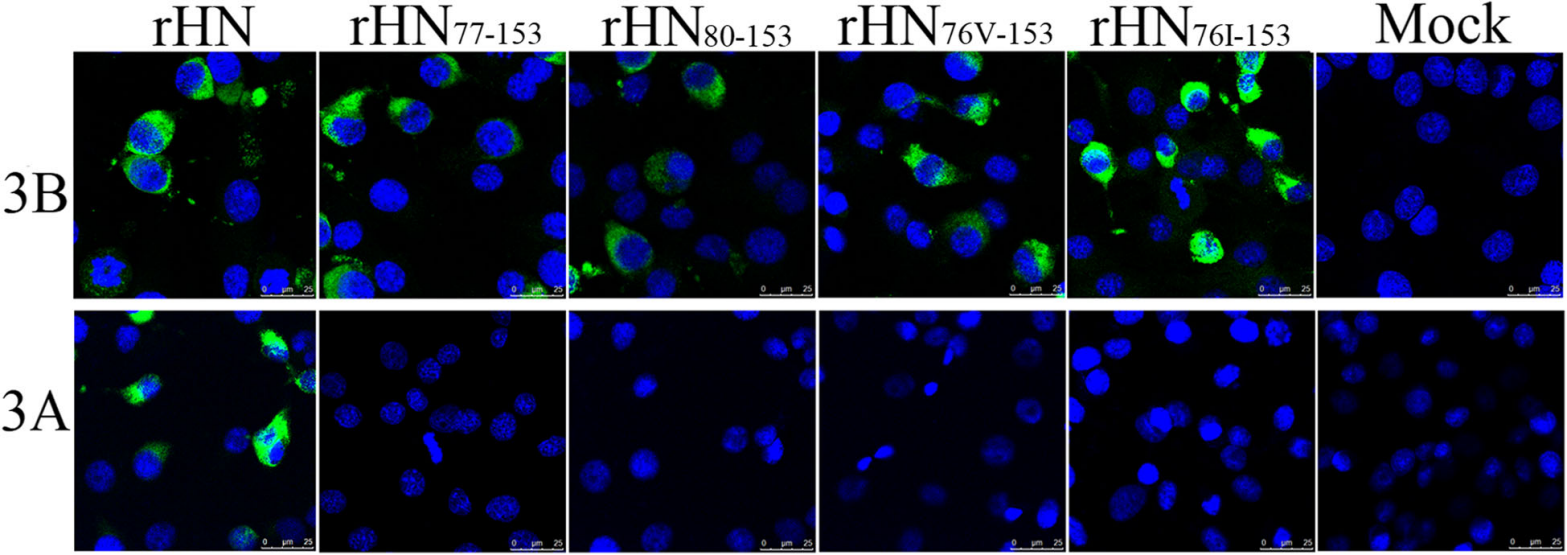
Analysis of the 3A mutant viruses by immunofluorescence. Confluent BHK-21 cells were infected with the wt or mutant viruses. After 6 h incubation at 37 °C, the cell were fixed and stained with FMDV specific anti-3A or anti-3B MAb, followed by incubation with fluorescein isothiocyanate (FITC)-conjugated secondary antibody. The cells were visualized under a laser-scanning confocal microscope (LSCM, Leica SP8). Bar, 25 μm

**Fig. 4:**
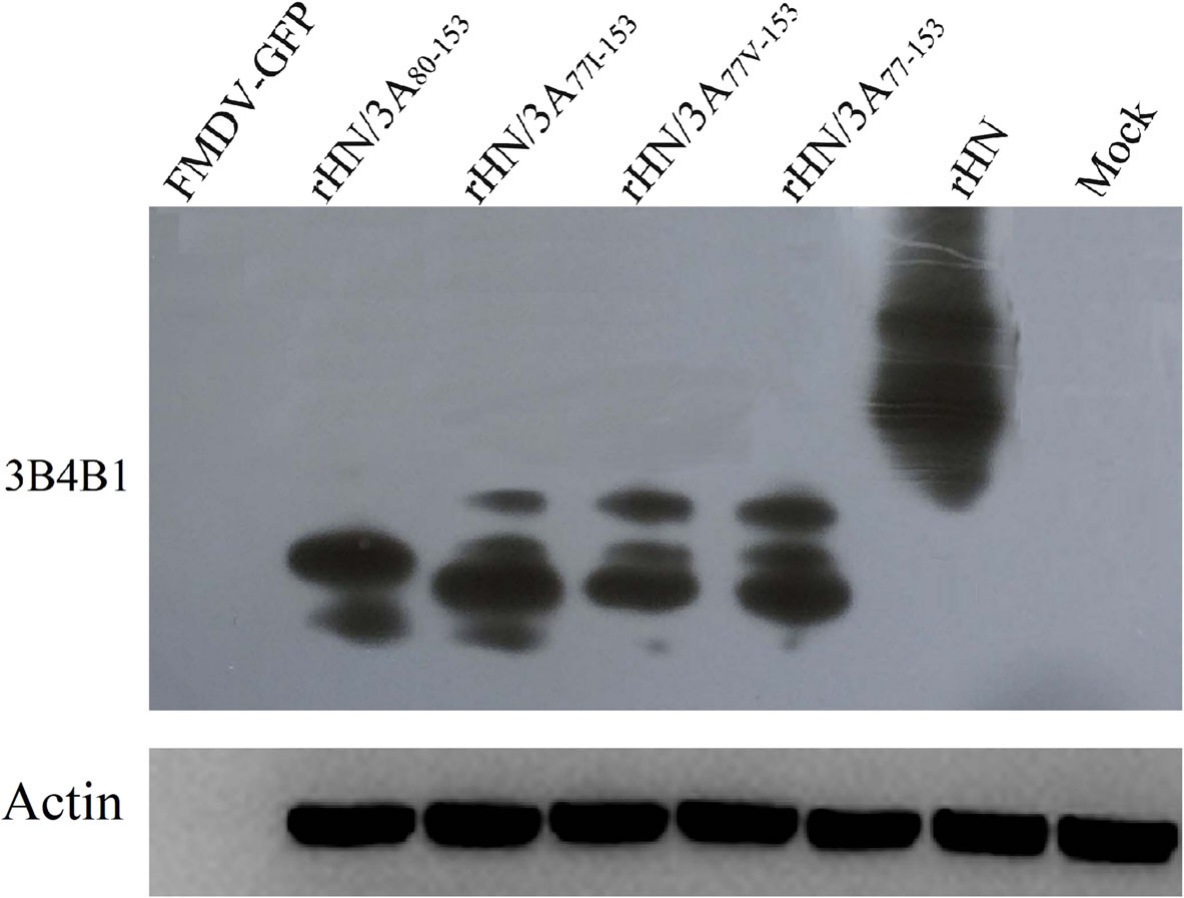
Analysis of the 3A mutant viruses by western blotting. BHK-21 cells were infected with the wt or mutant viruses. After 8 h incubation at 37 °C, cell extracts were prepared and proteins were separated on a 12% SDS-PAGE, blotted, and probed with primary antibodies (FMDV 3B monoclonal antibody and anti-GFP antibody) and secondary antibody (HRP-conjugated goat anti-mouse IgG) respectively

In order to observe the antigenic profile of the mutant and wt viruses, virus-infected cells were examined by immunofluorescence assay and western blotting with anti-FMDV 3A and/or 3B protein-specific monoclonal antibody, respectively. The results showed that all cells infected with the mutant and wt viruses were immunoreactive with FMDV 3B MAb and displayed 3B protein expression (Fig. 3 and Fig. 4), in contrast, only the wt virus was immunoreactive with 3A MAb targeted aa 109–115 of 3A protein, while both r/HN/3A_77–153_ and r/HN/3A_80–153_ viruses failed to react against 3A MAb (Fig. 3), indicating the deletions of aa 77–153 and 80–153 of 3A affected the ability of the mutant FMDVs to be recognized by 3A MAb but not by 3B MAb.

### The fusion of eGFP to the C terminus of 3A prevents vitro translation of the mutant full length RNAs

Previous studies showed that the recombinant viruses expressing the green fluorescent protein (GFP) are valuable for the detection of virus replication in vitro and the study of virus pathogenesis in vivo. To better investigate FMDV life cycle and evaluate if 3A could accept foreign gene in the largest nonessential region, eGFP gene was inserted into one of the mutant clone (pOFS/3A_77–153_). Then, the mutant clone was transfected into BSR/T7 cells. eGFP autofluorescence was not detected at 6, 12, 24 and 48 h post-transfection, which is different from previous study that the insertion of the eGFP ORF into the FMDV genome (the junction between P1 and 2A) imposed replicon-like characteristics [23]. Three additional passages were performed in the BHK-21cells and CPE was not appeared during these passages. We also can not detect eGFP and 3B protein expression from the infeced cells with the transfected supernatant by immunofluorescence (data not shown) or western blot (Fig. 4). These results possiblly suggest that the fusion of the GFP gene to the C-terminus of 3A had a negative effect on vitro translation of the mutant full length RNAs and the production of infectious viruses, however, the mechanism by which is unknown.

### The substitution of the residue 76 of 3A has no effect on the production of infectious FMDV

**Fig. 5:**
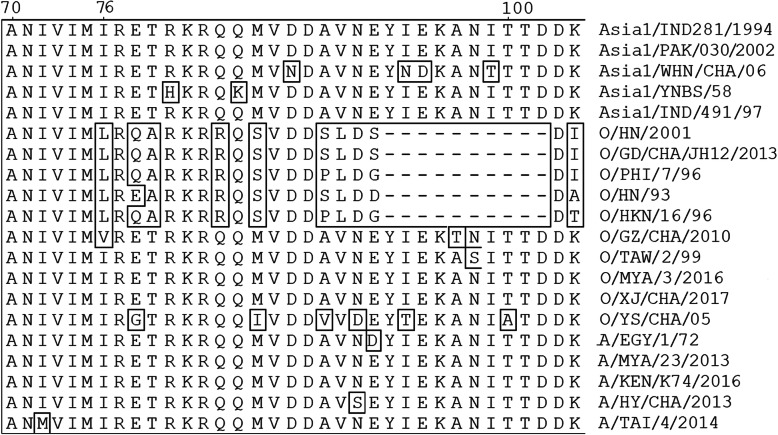
Alignment of the amino acid sequence of FMDV non-structural protein 3A: Asia1/IND/281/1994 (MF372126.1), Asia1/PAK/030/2002 (JF749849.1), Asia1/WHN/CHA/06 (FJ906802.1), Asia1/YNBS/58 (AY390432), Asia1/IND/491/97 (AY687334.1), O/HN/2001 (EU400597.1), O/GD/CHA/JH12/2013 (KU204894.1), O/PHI/7/96 (AJ294999.1), O/HN/CHA/93 (Li et al.,2012), O/HKN/16/96 (AJ294996.1), O/GZ/CHA/2010 (JN998086.1), O/TAW/2/99 (AJ295000.1), O/MYA/3/2016 (MG983703.1), O/XJ/CHA/2017 (MF461724.1), O/YS/CHA/05 (HM008917.1), A/EGY/1/72 (MH053305.1), A/MYA/23/2013(KY322678.1), A/KEN/K74/2016 (MN116688.1), A/HY/CHA/2013(KT968663), A/TAI/4/2014(KY322679.1). O/HN/CHA/93 is the parental virus of the genetically engineered FMDV r/HN

**Fig. 6:**
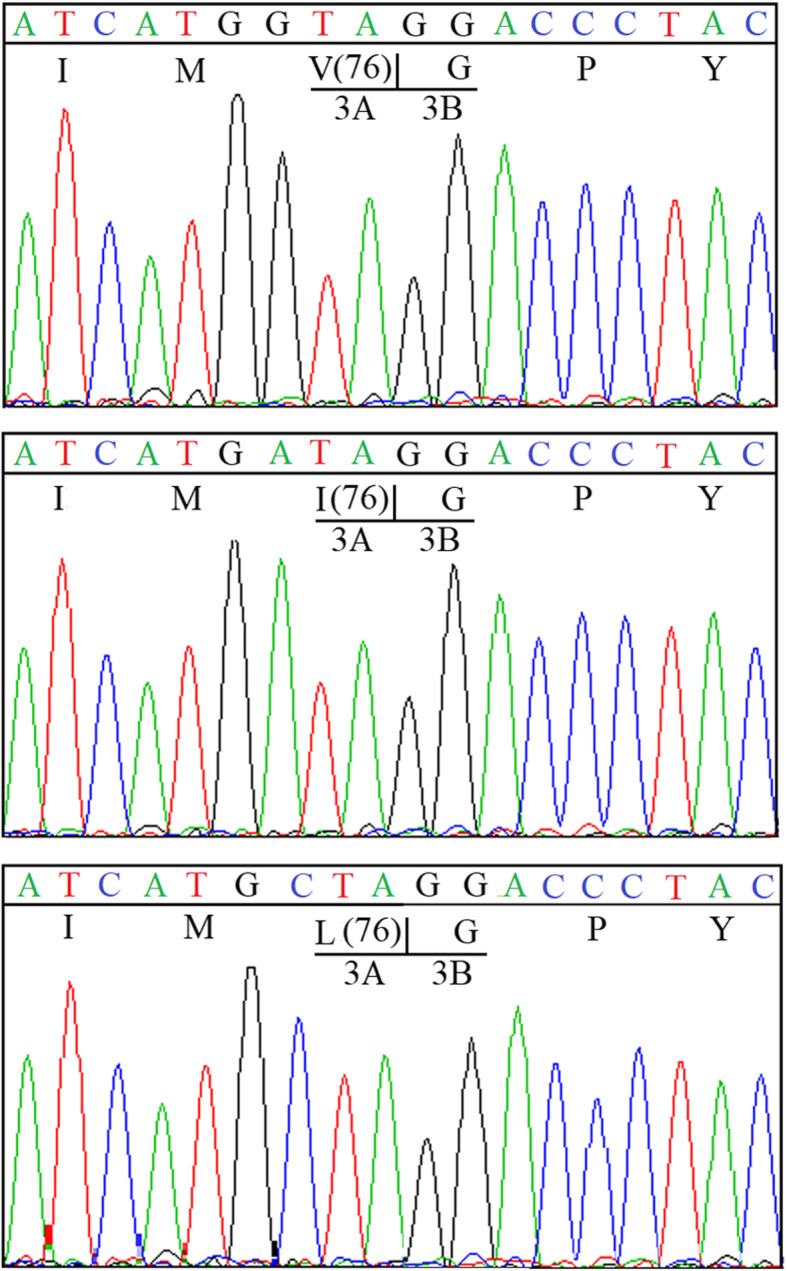
Sequencing chromatogram of the supernatants from transfected cells. The cell culture supernatants from transfected cells were harvested and RNAs were extracted with viral RNA mini kit. The cDNA fragments containing 3A were amplified by RT-PCR and sequenced an ABI 3700 sequencer

Multiple sequence alignment of FMDV 3A proteins indicated that the residue 76 L of 3A is highly conserved among the isolates of different serotype (Fig. 5), especially for the isolates of Cathay topotype (34 isolates of Cathay topotype all have the residue 76 L in 3A protein (Data not shown)). To determine whether the residue 76 L of 3A of FMDV O/HN/CHA/93 (Cathay topotype) would be replaced by valine (V) and isoleucine (I) which are found at same position in the isolates of the other topotype. The substitutions of 3A L76V and 3A L76I were introduced into one of the 3a deletion mutant by overlapping PCR. The mutant full-length plasmids were transfected into BSR/T7 cells, the viable viruses were successfully rescued. RT-PCR (Fig. 2) and sequencing (Fig. 6) confirmed the presence of the corresponding deletions and substitutions. These results revealed that the substitutions of L76V and L76I in 3A do not affect virus viability.

### The genetic stability of the 3A mutant viruses

To investigate the genetic stability of the mutant viruses, all viruses were serially passaged five times in BHK-21 cells, supernatants of each passage were collected and the cDNA fragments containing 3A were amplified by RT-PCR and sequencing. The results demonstrated that all the viruses retained the targeted deletions and substitutions and had no other mutations within 3A (data not shown). To further confirm whether additional compensatory mutations were present in other genomic regions, a total of four overlapping cDNA fragments of FMDV were obtained by RT-PCR, followed by nucleotide sequencing. Our data revealed that there were no other detectable mutations in the 3A mutant viruses except for virus r/HN/3A_76I-153_. This virus acquired the substitutions of G38S and E50Q in 3C non-structural protein during the process of passage, which are highly conserved in other isolate of type A, O and Asia1.

### Growth property of the 3A mutant viruses

**Fig. 7:**
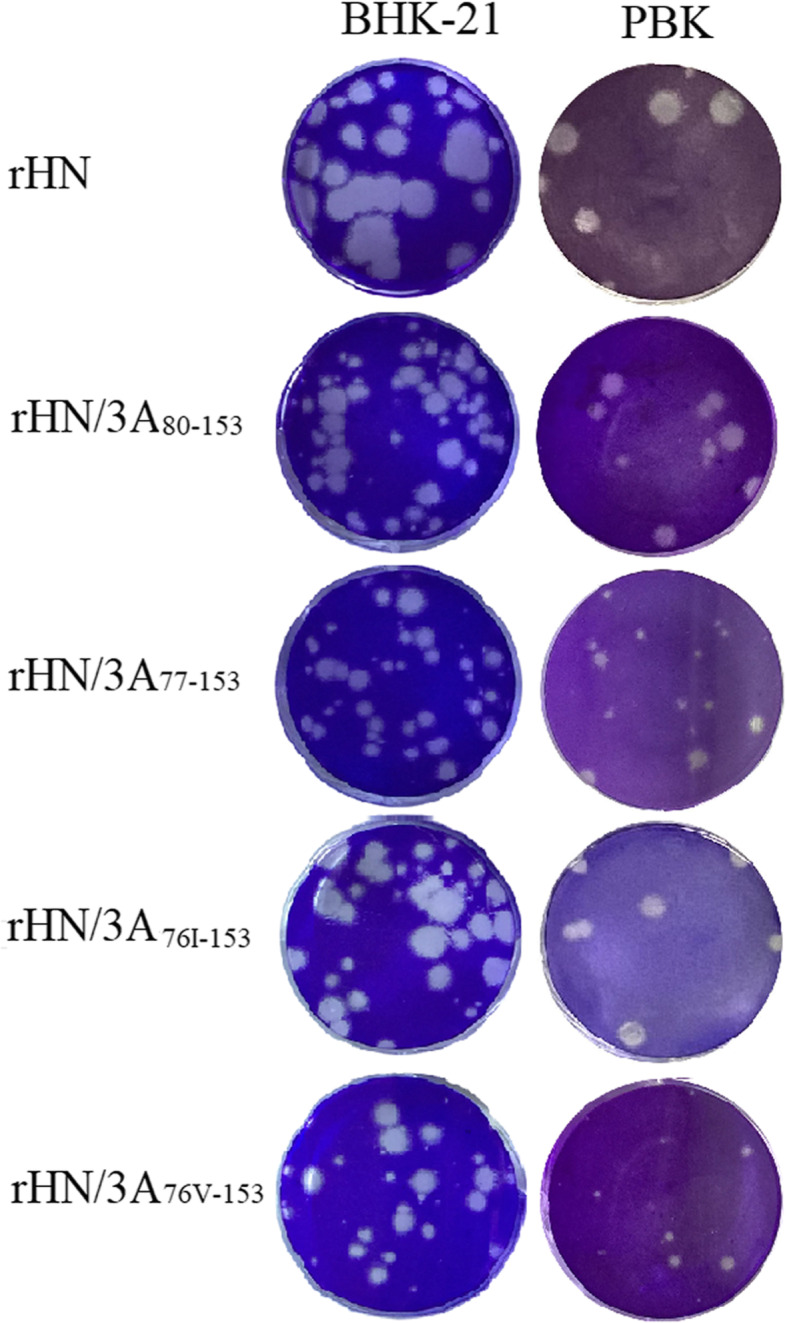
Plaque morphology of the wt and 3A mutant viruses in BHK-21 and FPK. Cells infected with with r/HN, r/HN/3A_77–153_, r/HN/3A_80–153_, r/HN/3A_76I-153_ and r/HN/3A_76V-153_ were incubated for 1 h at 37 °C, and after adsorption, 0.6% gum tragacanth overlay was added, and then monolayers were stained with crystal violet at 48 h post-infection

**Fig. 8:**
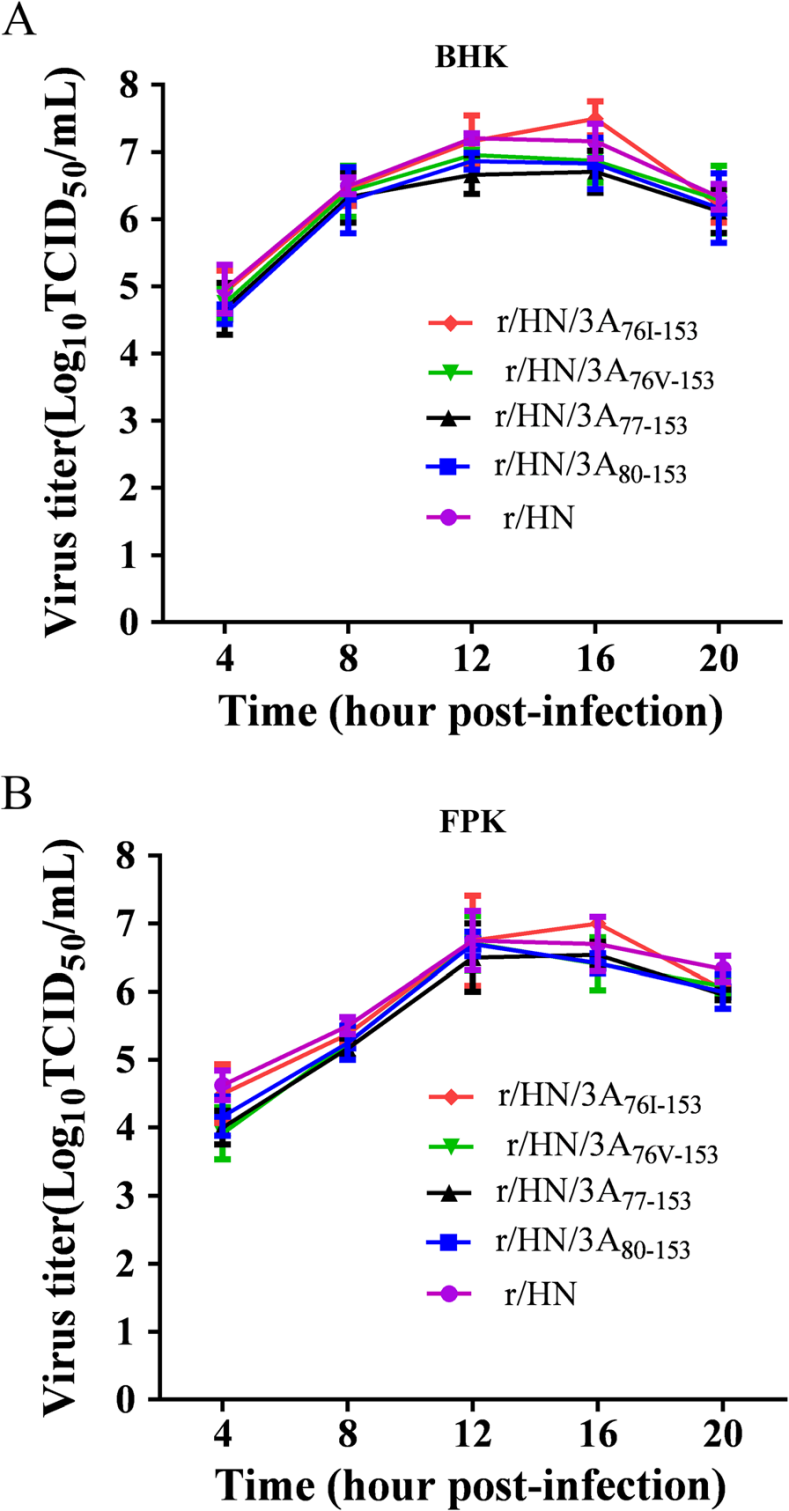
One-step growth curves of the wt and 3A mutant viruses in cells. Monolayer cells were infected with r/HN, r/HN/3A_77–153_, r/HN/3A_80–153_, r/HN/3A_76I-153_ and r/HN/3A_76V-153_ at an Multiplicity of infection (MOI) of 1 PFU/cell. At 4, 8, 12, 16 and 20 h post-infection, the supernatants were harvested and the virus titers were determined by TCID_50_/mL on BHK-21(A) and FPK(B) cells. The values of the viral titers represent the average obtained from triplicate experiments

To determine the effect of the deletions and substitutions in 3A on FMDV replication and preliminarily evaluate the species-specific tropism of the mutant viruses, the ability of the mutant and wt viruses to replicate in BHK-21 and primary fetal kidney cells (FPK and FBK) were first examined by viral plaques. The results showed that all the rescued viruses produced smaller plaques in BHK-21 and FPK cells compared with the wt virus r/HN (Fig. 7) and were completely unable to form any visible plaques in FBK cells (data not show), indicating that the deletions and substitutions affect viral plaque phenotype, but not change the ability of viral replication in primary cell. The virus r/HN/3A_76v-153_ formed plaques smaller than those formed by the virus r/HN/3A_77I-153_, but similar to those of the parental virus (r/HN/3A_77–153_) (Fig. 7). The ability of these viruses to replicate in BHK-21 and FPK cells was further analyzed by one-step growth kinetics, the results demonstrated that the rescued viruses share similar growth characteristics in BHK-21 and FPK cells except that the virus r/HN/3A_76I-153_ had significantly increased viral titers in BHK-21 cells after 16 h post-infection (Fig. 8a and Fig. 8b). The virus r/HN/3A_76I-153_ with higher replication capacity could be the candidates for marker vaccine in the future. The difference of growth and plaque phenotype of the virus r/HN/3A_76I-153_ still needs further determine whether the substitution(L76I) or the substitution (L76I) combined with the mutation of 38S and 50Q in 3C account for it.

## Discussion

The FMDV 3A protein is a multifunctional protein involved in many aspects of viral life-cycle. The N-terminal portion (aa 1–76) of the 3A protein is highly conserved and few mutations were found in this region, implicating that the N-terminal portion of 3A is essential for viral RNA replication, which has been certified by construction of several N-terminal deletion mutants of 3A [18]. However, the C-terminus of the 3A protein (aa 77–153) has been shown to exhibit considerable variability in size and nucleotide sequence, while it plays critical role in the viral RNA synthesis in FBK cells [14]. The highly genetic variability of 3A protein strongly suggests that this region may plays a very limited role for viral production. Accordingly, using reverse genetics technique, we demonstrated that this hypothesis is correct, the aa 77–153 rather than aa 81–153 of 3A was non-essential for production of infectious FMDV and the deletion did not obviously affect viral RNA replication, but affect viral plaque phenotype. Additionally, we also indicated that the deletion of the the residue 76 is lethal to the virus, suggesting the integrity of hydrophobic domain is vital for the completion the life cycle of FMDV. Although the deletion of aa 76–153 in 3A protein affect production of viable FMDV, the conserved residue 76 L in 3A of FMDV of Cathay topotype could be replaced by valine (V) and isoleucine (I) found in other isolates of different topotype and the substitutions did not affect the production of infectious viral particles. The virus carrying 3A L76V displayed similar growth kinetics and plaque phenotype with the parental virus, while the virus owning 3A L76I had larger plaque and a slightly higher viral titer in BHK-21 cell at16h post-infection. This virus obtained additional mutation (G38S and E50Q of 3C) during the process of passage in BHK-21 cells. Whether the single substitution of 3A L76I or the substitution of 3A L76I combined with 38S and 50Q in 3C account for the difference of the growth and plaque phenotype still needs further study.

Previous study indicated that a single amino acid substitution in 3A protein can mediate adaptation of foot-and-mouth disease virus to the guinea pig [24]. The species-specific tropism of the mutant viruses were preliminarily assessed in FBK and FPK, the results demonstrated that the mutant viruses have similar plaque forming ability to that of the wt virus and the substitution of L76I and L76V in 3A did not change species-specific tropism in primary cells.

FMDV as a potential viral vector expressing foreign gene and the introduction of the reporter genes (such as green fluorescent protein (GFP) and renilla luciferase (RL)) into RNA viruses to further characterize virus life cycle have been numerously reported [23, 25–31]. For FMDV, the insertion sites of small foreign gene (not more than 20aa) were mainly focused on the intergenic regions of FMDV structural and non-structural proteins [25–27] and larger foreign gene (GFP and RL) were introduced into the junction between P1 and 2A [23, 27]. However, the viable FMDVs expressing reporter proteins were difficult to rescue or generally unstable [23, 27], which pose the obstacle on the study of FMDV replication in vitro and pathogensis in vivo as well as rapid screening of antiviral drugs. To explore the genetic flexibility of the FMDV 3A protein, the eGFP was introduced into the nonessential regions of 3A. The result showed that eGFP autofluorescence was not detected after transfection. Accordingly, eGFP fluorescence and 3B protein also can not be found from the infeced cells with the transfected supernatant by immunofluorescence and western blot. The possible reason is that the insertion of the eGFP gene into 3A might exert a strong structural influence on surrounding sequences, which lead to prevent vitro translation of the mutant full length RNAs, but the mechanism is poor understand. Much investigations should be further made to study the ability of the non-essential regions of 3A accept foreign genes.

## Conclusions

This study firstly reported that the FMDV could tolerate the deletion of 77–153 aa in 3A protein without affecting production of infectious viral particle in cultured cells. Such nonessential region will provide ideal insertion site for investigating the capacity of FMD viral vector expressing foreign gene and the mutant FMDVs could be the potential candidates for genetic marker vaccine.

## Data Availability

All data generated or analyzed during this study are included in this published article.

## Abbreviations

FMDV: Foot-and-mouth disease virus
FMD: Foot-and-mouth disease
L: Leucine
V: Valine
I: Isoleucine
BHK: Baby Hamster Kidney
RT-PCR: Reverse transcription polymerase chain reaction
SATs: South African Territories
HR: Hydrophobic domain
DMEM: Dulbecco’s Modified Eagle Medium
FCS: Fetal calf serum
GMEM: Glasgow minimal essential medium
wt: Wild-type
CPE: Cytopathic effect
FITC: Fluorescein isothiocyanate
Mab: Monoclonal antibody,
DAPI: 4′, 6-diamidino-2-phenylindole
PVDV: Polyvinylidene fluoride
HRP: Horseradish peroxidase
PBS: Phosphate-buffered saline
TCID_50_: Tissue culture infective dose
